# Development and validation of sex-associated markers using whole-genome re-sequencing in frog *Quasipaa spinosa*

**DOI:** 10.3389/fgene.2025.1596192

**Published:** 2025-06-02

**Authors:** Liaoruilin Zhang, Juan Li, Xiang Li, Jinrong He, Jie Zhou, Jinliang Hou, Yulu Liu, Lei Zhang, Yanfei Huang, Hong Li, Xiaolin Liao, Xinhua Liu, Yazhou Hu, Deliang Li, Jianguo Xiang

**Affiliations:** ^1^ Fisheries College, Hunan Agricultural University, Changsha, Hunan, China; ^2^ Animal Husbandry and Fisheries Affairs Center, Leiyang, Hunan, China

**Keywords:** *Quasipaa spinosa*, whole-genome re-sequencing, genome-wide association study, INDEL, sex-associated marker

## Abstract

The development of sex markers is crucial for addressing monosexual breeding in aquaculture species and for identifying traits that are sexually inherited, especially for elucidating the mechanisms of sex determination in amphibians. In aquaculture, comprehending sex determination is especially vital because the market value of animal products frequently depends on their sex. *Quasipaa spinosa* (Anura, Dicroglossidea) is a valuable frog species in the aquaculture industry of China and southeast Asia, yet there exists limited genomic information regarding this organism. Current data indicates that the adoption of all-male breeding techniques in *Q. spinosa* could substantially benefit the Chinese aquaculture industry, both by augmenting its economic prospects and by ensuring the effectiveness of wildlife reintroduction efforts. The growth rate, adult size, disease resistance, and other traits of male *Q. spinosa* surpass those of females, making the development of all-male breeding a significant focus in the *Q. spinosa* aquaculture industry. Therefore, it is imperative to establish a marker specific to males. In this research, we used the male *Q. spinosa* genome as reference and performed whole-genome resequencing on 30 males and 30 females. Subsequently, we exhibited evident sexual differentiation on chromosome 3 and primers were designed for PCR detection of the identified candidate male INDEL loci. Ultimately, two sex-associated INDELs that could be effectively detected were obtained and validated on the samples collected from the remaining three locations, thereby confirming the robustness of these two INDELs for sex identification in *Q. spinosa*.

## 1 Introduction

To date, all studied amphibian species have exhibited genetic sex determination (GSD) (Ruiz et al., 2024). However, in certain cases, epigenetic factors such as temperature can override GSD, leading to sex reversal (Schmid et al., 2001) or a biased sex ratio (Lambert et al., 2018). Nevertheless, GSD predominantly governs natural populations (Berset-Brandli et al., 2006). According to cytogenetic analysis, several GSD amphibians can be distinguished by heteromorphic sex chromosomes. For instance, *Gastrotheca riobambae* (Schmid et al., 1983) and *Fejervarya limnocharis* (Patawang et al., 2014) exhibited male heterogamety (XX/XY), while *Pyxicephalus adspersus* (Schmid and Bachmann, 1981) and *Physalaemu epididier* (Nascimento et al., 2010) displayed female heterogamety (ZZ/ZW). Moreover, sex-linked markers could also differentiate GSD amphibians, with *Quasipaa Boulengeri* (Yang et al., 2022) demonstrating the XX/XY type and *Xenopus laevis* exhibiting the ZZ/ZW (Yoshimoto and Ito, 2011) or ZWY type (Furman et al., 2020). Nevertheless, the majority of anurans exhibited homomorphic sex chromosomes (Ma and Veltsos, 2021), necessitating the development of sex markers for distinguishing GSD.

Many scholars had successfully developed sex markers in Amphibia using molecular techniques. For instance, restriction-site associated DNA sequencing (RADseq) to identify sex-specific markers in *Bufo bufo* (Nemeshazi et al., 2022) and RADseq employed to analyze a large dataset of 929 African clawed frogs, successfully identifying sex-linked SNPs, sex chromosomes, and elucidating the origin of sex chromosomes as sex-determination loci established prior to recombination suppression (Evans et al., 2024). Similarly, RADseq revealed frequent sex-chromosome turnovers in true frog (Jeffries et al., 2018). Diversity arrays technology sequencing (DArTseq) identified 13 sex-linked SNPs and 8 male-linked loci in which two alleles from the same locus showed partial high sequence homology to *Dmrt1* in *Rana clamitans* (Lambert et al., 2016), while *Odorrana utsunomiyaorum* was found to be female heterogamety (Katsumi et al., 2022). Genotyping-by-sequencing (GBS) was employed for genotyping *Amolops mantzorum* (Luo et al., 2020) and confirming an XX/XY system in *Q. boulengeri* (Yang et al., 2022). Amplified fragment-length polymorphisms (AFLP) revealed that Palearctic green toads exhibited male heterogamety (Stock et al., 2011). Additionally, target region amplification polymorphism (TRAP) screened two male-linked sex markers in *R*. *dybowskii* (Xu et al., 2022). Quantitative Real-time PCR (qPCR) detected the ovarian early differentiation marker aromatase (*Cyp19*) in *Lithobates sylvaticus* (Navarro-Martin et al., 2012). However, no studies had yet reported sex markers for *Quasipaa spinosa*.

*Quasipaa spinosa* (Anura, Dicroglossidea) predominantly found in the hilly terrains of southern China and the hilly regions of northern Vietnam (Long et al., 2021) which was a valuable economic amphibian species with high nutritional value and served as a common source of medicine and food (Mei et al., 2018). However, owing to excessive capture, widespread application of chemical pesticides, and habitats loss, there has been a sharp decline in wild populations of this species (Liang et al., 2013). Consequently, both the International Union for Conservation of Nature (IUCN) Red List and the Chinese Red List of Species have listed it as a species at risk (Chan et al., 2014). *Quasipaa spinosa* displays pronounced sexual dimorphism in growth, with males exhibiting significantly higher rates of growth and body weight compared to females (Zhang et al., 2023). The implementation of all-male reproduction in *Q. spinosa* could not only increase its economic value but also help mitigate overexploitation of wild populations, thereby contributing to the conservation of biodiversity in Chinese natural habitats. However, similar to most anurans, *Q. spinosa* exhibited homomorphic sex chromosomes, making it challenging to accurately determine GSD (Zhang et al., 2024). Consequently, developing sex markers for *Q. spinosa* would facilitate advancements in genetic breeding and sex control techniques within the aquaculture industry.

This research utilized whole-genome re-sequencing techniques to develop two male molecular associated markers in *Q. spinosa*. Our research findings will contribute to the advancement of genome-scale breeding strategies for *Q. spinosa* and establish a theoretical groundwork for establishing a robust sex-determination system in *Q. spinosa*.

## 2 Materials and methods

### 2.1 Sample collection and ethics statement

A total of 60 *Q. spinosa*, consisting of 30 males and 30 females, were collected from a Shimen farm located in Hunan Province, China, for the purpose of conducting extensive whole-genome re-sequencing. The remaining trio of populations gathered from Changde (15 males and 18 females), Pingxiang (15 males and 14 females) and Changsha (20 males and 17 females) were utilized to validate sex-associated markers. Upon identifying the sex through the examination of black bursa in the chest and gonads, the leg muscles were harvested and preserved at −80°C for later DNA extraction. The research involving *Q. spinosa* and its methodology strictly followed the set ethical standards by the Animal Protection Committee (APC) of Hunan Agricultural University (ethics license: No. LSK 2024-D110).

### 2.2 DNA extraction and re-sequencing

DNA was extracted by Animal Genomic DNA Extraction Kit (Biosharp, Heifei, China) from leg muscles following pre-grind in liquid nitrogen. Qubit dsDNA HS Assay Kit (Sangon, Shanghai, China) was used to test concentration and 1% agarose gel electrophoresis to confirm integrity. Sangon Biotech (Shanghai) Co., Ltd. completed the preparation and re-sequencing of the library. Initially, Covaris (Woburn, United States) randomly fragmented 500 ng of measured DNA. Subsequently, the subsequent step involved the use of the Hieff NGS^®^ MaxUp II DNA Library Prep Kit for Illumina^®^ (YEASEN, Shanghai, China). Briefly, endprep enzyme was added to repair end and ligate A tail to 3′end. Subsequently, the adaptor was affixed utilizing an enhancer in conjunction with Fast T4 DNA ligase. The index primer was integrated through PCR, followed by the isolation of the amplified 400 bp product using DNA selection beads. The dimensions and concentration of the library were verified using Qubit 4.0 (Thermo, Waltham, United States) and 2% agarose gel electrophoresis, in that order. Ultimately, the libraries were combined and processed using a Novaseq6000 (Illumina, San Diego, United States) sequencer, employing a 2 × 150 bp paired end sequence kit as per the guidelines provided by the manufacturer.

### 2.3 Data analysis and sex-associated region in *Q. spinosa*

Unprocessed sequences with adaptors and bases of uncertain or inferior quality at the start or finish were excised using Fastp (https://github.com/OpenGene/fastp). Qualified sequences from each specimen were matched to the compiled reference genome (Hu et al., 2022) utilizing BWA v0.7.17 (Li, 2013), employing standard settings. Repetitive reads were eliminated, and the coverage metrics were determined through the use of SAMtools v1.9 (Li et al., 2009). The initial identification of variations was performed utilizing the GATK (Genome Analysis ToolKit, v4.1.2) (McKenna et al., 2010). SnpEff v4.3t (Cingolani et al., 2012) was used to finalize the functional annotation of every genetic variant. And the Circos plots were created by using R-circlize (Gu et al., 2014). Using VCFtools v0.1.16 (Danecek et al., 2011), SNPs and INDELs in VCF files underwent quality filtering to eliminate variants exhibiting poor quality and a missing rate exceeding 0.8. SNPs and INDELs underwent additional filtration via PLINK v2.0 (Chang et al., 2015). The process involved filling in absent genotypes with Beagle v5.4 (Browning et al., 2018), then re-filtering SNPs and INDELs via PLINK 2.0 to identify superior common SNPs and INDELs for additional examination (minor allele frequency >0.05; SNPs and INDELs in Hardy-Weinberg equilibrium <0.01; missing rate <0.15). The GWAS (Genome-wide association studies) for sexual phenotype was conducted using GLM (Generalized linear model) (Price et al., 2006), MLM (Mixed linear model) (Yu et al., 2006), and FarmCPU (Fixed and random model circulating probability unification) (Liu et al., 2016) in rMVP v3.6.0 (Yin et al., 2021), focusing on filtered SNPs/INDELs.

### 2.4 Validation of sex-associated markers

Primer 3 Plus (Untergasser et al., 2012) was utilized to develop the primers INDELs located on the sex-associated region sequence 78,403,285 and 296,192,082 of Chr 3. And their validity was confirmed in three populations: Changde, Pingxiang, and Changsha population by PCR. The PCR process involved 12.5 μL of 2 x Rapid Taq PCR Master Mix (comprising dNTPs, MgCl_2_, and buffer), along with 9.5 μL ddH_2_O, 1 μL each of forward and reverse primers, and 1 μL of template DNA. The procedure for amplification PCR proceeded in this manner: starting with denaturation at 95°C lasting 7 min, then 33 cycles of denaturation at 95°C for 45 s, annealing at Tm (Table 1) for 40 s, extension at 72°C for 45 s, and concluding with an elongation phase at 72°C for 7 min. The resulting PCR products were separated on a 2% agarose gel.

**TABLE 1:** The primer pairs of INDEL sex-associated markers.

Primer pair	Primer sequences (5′ to 3′)	Tm	Deletion	Position	Chromosome
Primer ξ	F: CCCATCCAAATGAAGCCCTT R: ATGTGGGCAGTTGCAGTC	56	23 bp	78,403,285	3
Primer δ	F: CCATCCACGTCAATGAGCAG R: TGCTAACTAGGCATGAGCGT	57	47 bp	296,192,082	3

## 3 Results

### 3.1 Whole-genome re-sequencing data analysis

**FIGURE 1:**
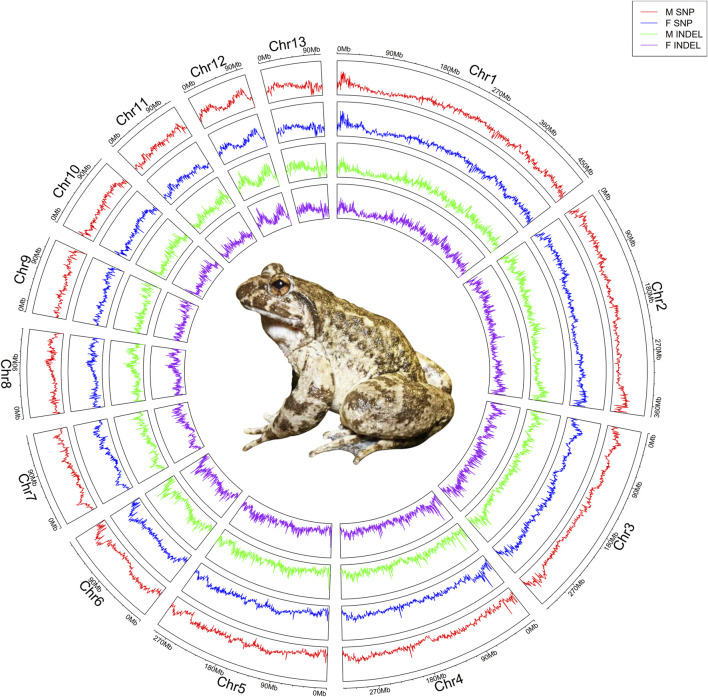
Revealing sex-associated variations in *Q. spinosa* by whole-genome re-sequencing. Re-sequencing information from 30 female and 30 male was juxtaposed with the reference genome, and the depth profiles of SNPs and INDELs were determined through a 100 kb sliding window, producing a single data point every 10 kb. The circular plot illustrated the genome-wide statistics of *Q. spinosa*, showcasing the depth of SNPs and INDELs across its 13 pairs of chromosomes from the outer to inner: male SNP, female SNP, male INDEL, and female INDEL. The center of the circle depicted an authentic image of *Q. spinosa*.

**FIGURE 2:**
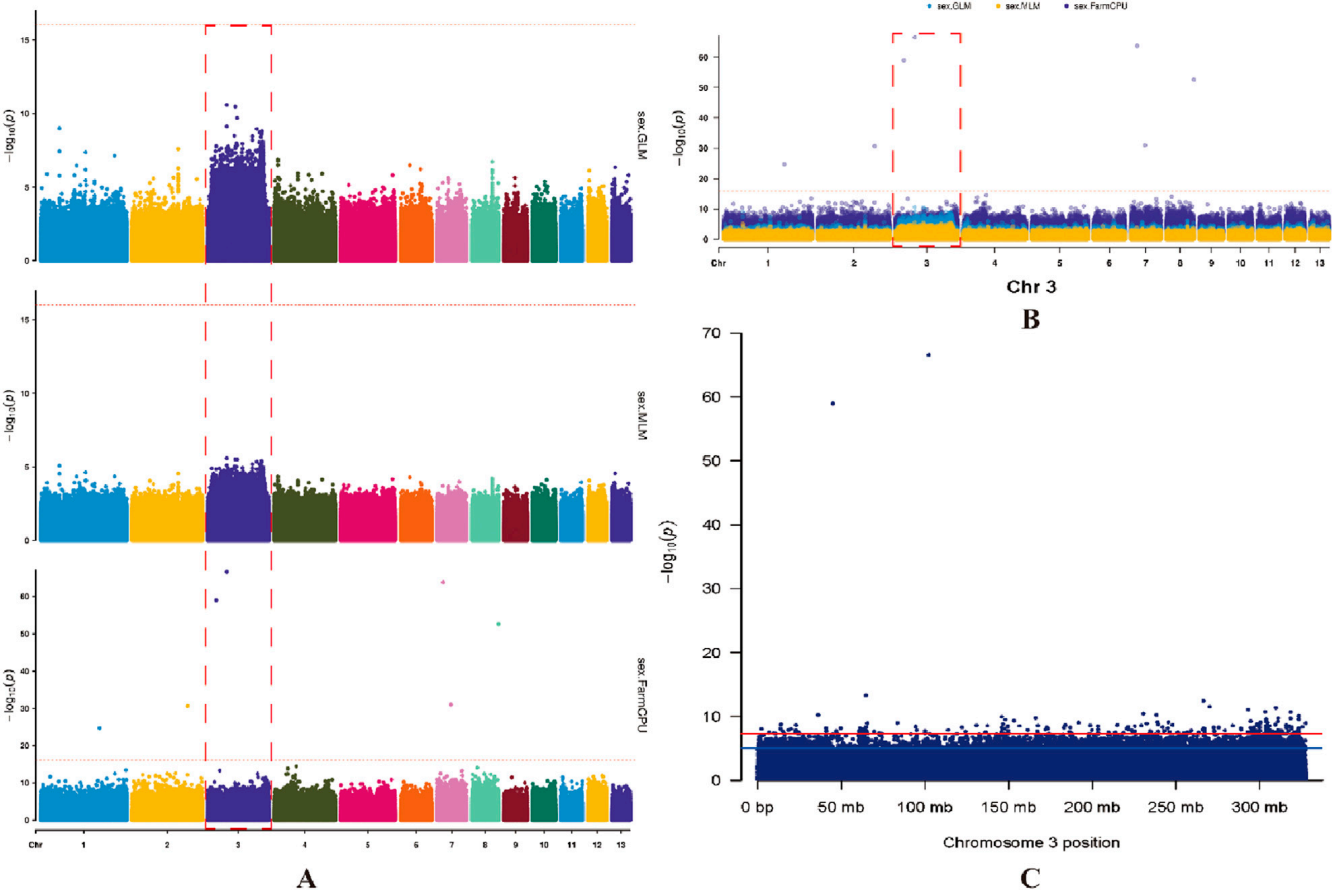
Sex difference SNP/InDel Manhattan plot in *Q. spinosa*. **(A)** the plots depict the association analysis for sex by GWAS: GLM, MLM, and FarmCPU, arranged from top to bottom. **(B)** comparison plot of three correlation analyses. **(C)** distribution of significant differences in SNPs on chromosome 3 with 10 kb resolution. A and B, the Y-axis represents -log_10_, which indicates the significant *p* value between the sexes. The X-axis denotes the chromosomes, with each number corresponding to one of the 13 pairs of chromosomes in *Q. spinosa*. A red dashed line, parallel to the X-axis, represents a vector indicating the smallest *p* value from all experiments. The red dashed box indicates the localization to chromosome 3 exhibiting a pronounced sex-associated difference. C, red line, parallel to the X-axis, corresponds to -log_10_(5e-8), while the blue line corresponds to -log_10_(1e-5).

The 30 male and 30 female individuals yielded a total of 7,428,976,654 and 7,784,167,862 clean reads, respectively, which exhibited an average coverage depth of 10× on the reference genome of the *Q. spinosa*. The BWA was employed to align the acquired sequences with the *Q. spinosa* reference genome, resulting in mapping rates of 99.72% for males and 99.69% for females. The overall genome coverage was 95.03% for males and 94.70% for females, and the average Q20 and Q30 values were 99.15% and 97.21% for males, while 98.77% and 96.54% for females. Moreover, the nucleotide analysis of the constructed scaffolds showed an average GC content of 45.53% in males and 45.09% for females (Supplementary Table S1). The genetic differences between females and males were characterized by aligning reads to a chromosomal reference genome, followed by the calculation of SNPs and INDELs for the entire genome through a comparison of whole-genome mapping in both genders (Figure 1). The MLM/GLM/FarmCPU model association analyses unveiled significant sex differences on chromosome 3, with a screening threshold of −log_10_(1e-5) (Figure 2). Specifically, the MLM identified 41,776 SNPs and 5,440 INDELs (Supplementary Table S2) and the GLM detected 17,606 SNPs and 2,212 INDELs (Supplementary Table S3), while the FarmCPU revealed 443 SNPs and 82 INDELs (Supplementary Table S4).

### 3.2 Location of sex-associated region and identification of the sex-associated markers

**FIGURE 3:**
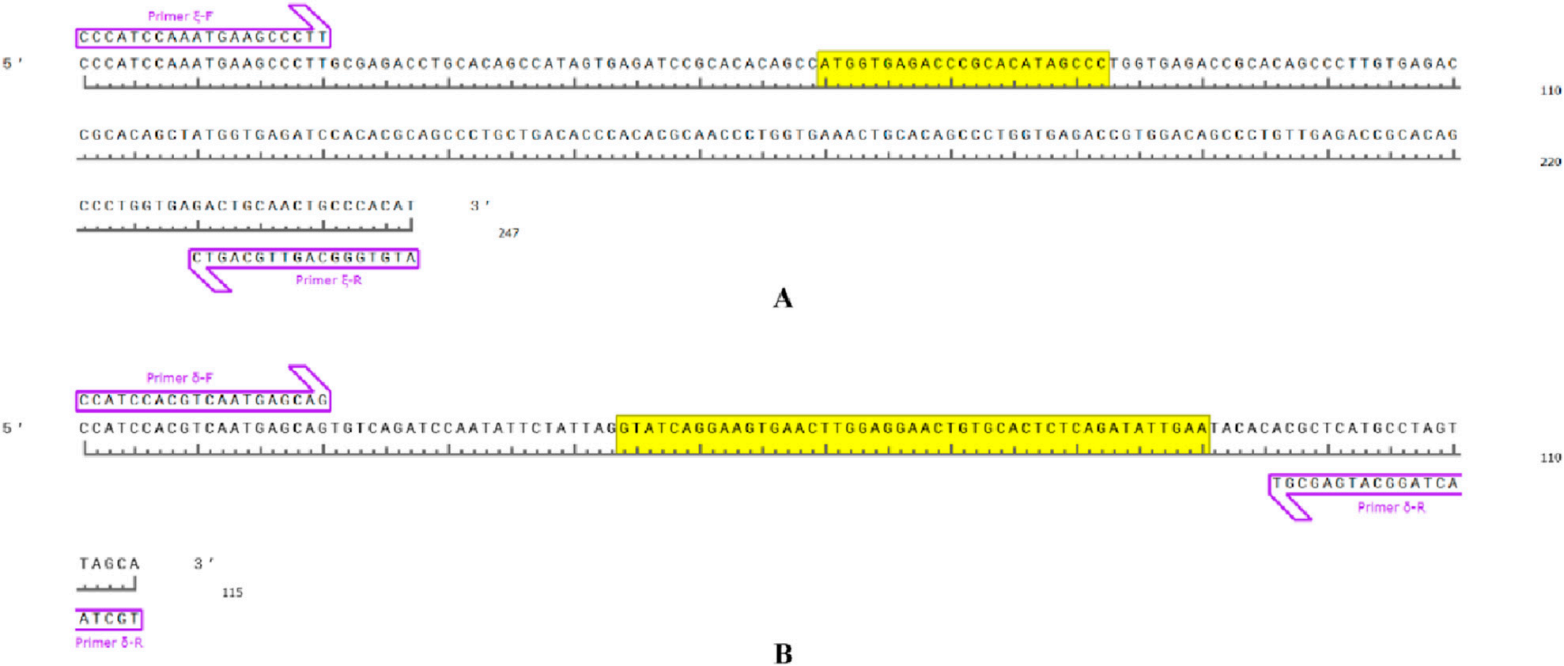
Sex-associated primer-amplified DNA fragments in *Q. spinosa* and the yellow base region corresponds to the DNA deletion fragment. **(A)** primer ξ amplification region. **(B)** primer δ amplification region.

**FIGURE 4:**
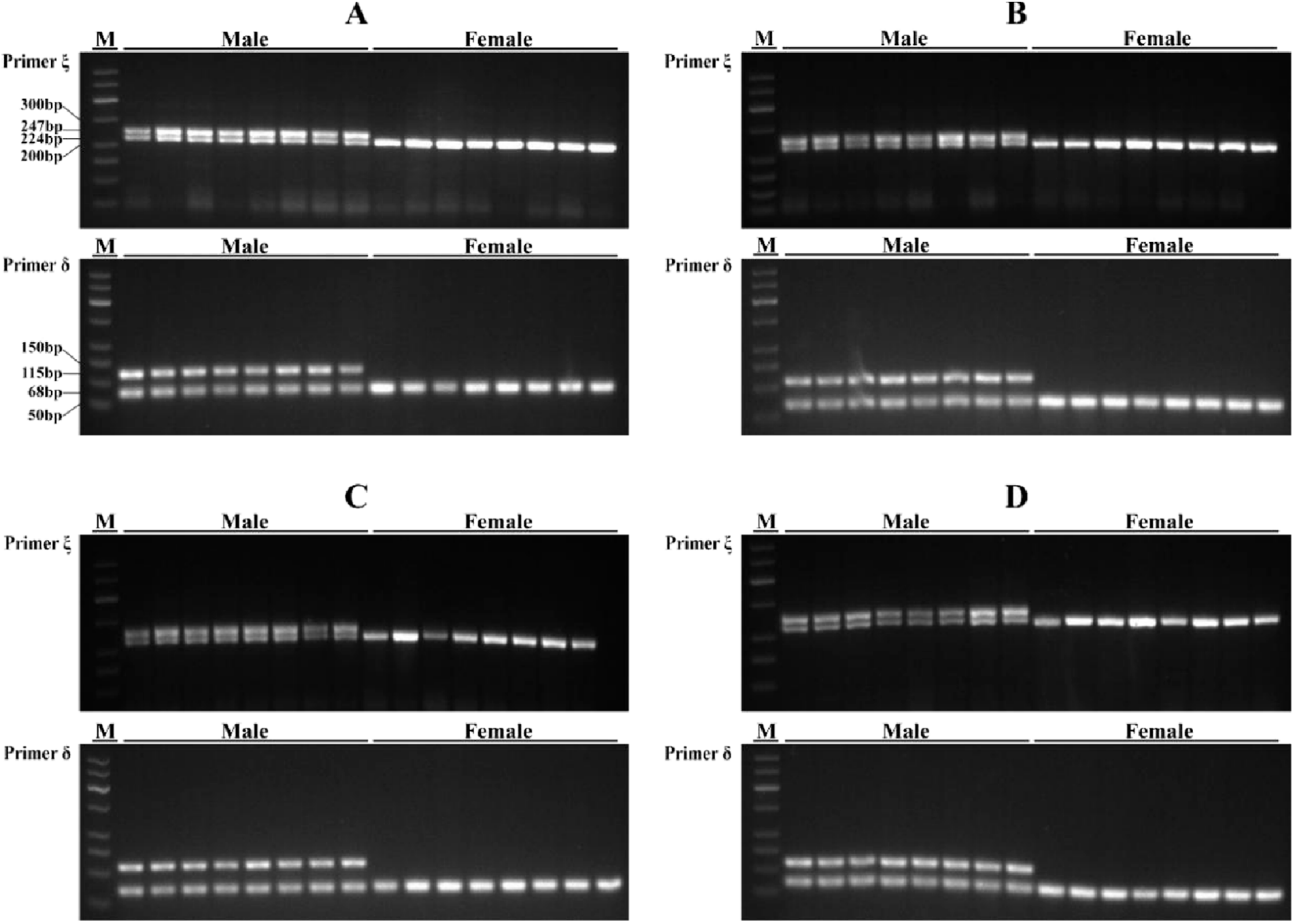
PCR amplification results of primer ξ and primer δ in four *Q. spinosa* populations. **(A)** Shimen population; **(B)** Changde population; **(C)** Pingxiang population; **(D)** Changsha population. M, DNA marker DL500.

By designing primers for 271 INDELs (from GLM analysis) sex-associated loci, PCR amplification and agarose gel electrophoresis revealed the successful amplification of two primer pairs in the Shimen population, yielding male-associated bands. The INDEL ξ was located at position 78,403,285 bp on chromosome 3 and involved a 23 bp deletion, while the INDEL δ was situated at position 296,192,082 bp on chromosome 3 with a 47 bp deletion. Both loci located within regulatory regions and were separated by a distance of 217,788,797 bp (Table 1; Figure 3). The two sex-associated markers were amplified using PCR to generate two bands for all males and a single band in all females. Primer ξ amplified bands of 247 bp and 224 bp in males and a band of 224 bp in females, while primer δ amplified bands of 115 bp and 68 bp in males and a band of 68 bp in females (Figure 4A). In addition, the PCR amplification of two primer pairs was conducted in the populations of Changde (Figure 4B), Pingxiang (Figure 4C) and Changsha (Figure 4D). The results demonstrated successful differences between male and female individuals.

## 4 Discussion

Sex markers played a pivotal role in streamlining sex-controlled breeding initiatives and in deciphering the complex molecular pathways implicated in both sex differentiation and determination (Chen et al., 2018). Whole-genome re-sequencing enabled the identification and analysis of multiple target genes, allowing for the investigation of their expression and regulation (Teng and Xiao, 2009). The employment of whole-genome resequencing facilitated the creation of numerous sex markers for application in aquaculture species in recent years, for example, *Leiocassis longirostris* (Luo et al., 2022), *Spinibarbus hollandi* (Huang et al., 2024), *Megalobrama amblycephala* (Wen et al., 2024), *Pelodiscus sinensis* (Zeng et al., 2024), *Protosalanx hyalocranius* (Xing et al., 2021), *Haliotis discus hannai Ino* (Luo et al., 2021), *Ambystoma mexicanum* (Keinath et al., 2018), *Oreochromis mossambicus* (Tao et al., 2022), *Oplegnathus punctatus* (Li et al., 2020), *Collichthys lucidus* (Xiao et al., 2020). The identification of sex-associated markers on chromosomes through reference genome mapping provided valuable insights into putative sex chromosome locations. In our research, we discovered 2,212 potential sex-associated INDELs and ultimately identified two sex-associated INDELs which effectively distinguished between sexes and which inferred that *Q. spinosa* exhibited male heterozygous system.

The process of pinpointing sex markers aided in the localization of genomic segments that dictated sex. This process ultimately uncovered genes that were involved in sex determination (Mascali et al., 2022). The regulation of gonadal differentiation in vertebrates involved genes such as *Dmrt1*, *Foxl2*, *Sox9*, *Cyp19*, all of which are highly conserved (Nakamura, 2013). In the current state of research, only one sex-determining gene, dmrt1-paralogue (dm-w) had been identified in female heterogamous African clawed frog (ZW/ZZ) among a total of 8,740 amphibian species and a recently discovered candidate major locus was believed to control male heterogamy (Kuhl et al., 2024). The failure of the two male-associated loci developed in *Q. spinosa* to recognize functional genes known to be sex-linked may be attributed to the hindrance caused by fragmentation of the assembled genome, which posed challenges for predicting these coding genes (Mascali et al., 2022). We proposed that further in-depth investigations, such as gonadal transcriptome analysis or more comprehensive genomic screening, were warranted to elucidate the mechanism of sex determination in *Q. spinosa*.

The prevalence of sexual dimorphism in aquatic organisms necessitated the implementation of unisex aquaculture, which could effectively extend the growth period, significantly enhanced aquaculture production, improved economic efficiency, and contributed to species conservation (Schimek et al., 2022; Song et al., 2021). Therefore, sex control represented a valuable undertaking in the realm of aquatic animals. The implementation of genetic breeding programs had been initiated in numerous countries and institutions with the aim of attaining unisexual populations (either all-male or all-female) or achieving high proportions of males or females, thereby enhancing the efficiency of aquaculture (Gui et al., 2022). However, it was crucial to note that species through single-sex breeding were prohibited from being released into the wild as this would disrupt ecological balance. The growth rate, disease resistance, and other traits of male *Q. spinosa* surpassed those of females in the adult stage, making the development of mono-male culture a focal point in the *Q. spinosa* aquaculture industry (Li et al., 2024). The market price of *Q. spinosa* in Chinese aquaculture industry had increased by 1.3 times from 2016 to 2020 (Zhou, 2020; Zhou et al., 2016), and the species necessitated a stringent farming environment that prohibited high-density cultivation which not only contributed to the advancement of the agricultural economy, but also facilitated the augmentation and released measures aimed at safeguarding wild resources (Zhang and Zhou, 2016). We had developed sex-associated markers using a simplified method to effectively identify distinguish sex in *Q. spinosa*, thereby facilitating research on nature conservation.

## 5 Conclusion

*Quasipaa spinosa* is an economically significant animal and a vulnerable species in the world. In this research, we accomplished the development of sex-associated markers for *Q. spinosa* through the screening of two sex-associated INDELs on chromosome 3 using whole-genome re-sequencing and GWAS analysis. These findings provide essential molecular genetic information for *Q. spinosa*, which is vital for advancing the research on sex determination mechanisms and for developing strategies for all-male breeding techniques.

## Data Availability

The original contributions presented in the study are publicly available. This data can be found here: 10.5281/zenodo.15524263; 10.5281/zenodo.15524265; 10.5281/zenodo.15524267; 10.5281/zenodo.15524269; 10.5281/zenodo.15524271; 10.5281/zenodo.15524273; 10.5281/zenodo.15524275; 10.5281/zenodo.15524277; 10.5281/zenodo.15524287; 10.5281/zenodo.15524289.
